# Improving Malawian teachers' mental health knowledge and attitudes: an integrated school mental health literacy approach

**DOI:** 10.1017/gmh.2014.8

**Published:** 2015-02-16

**Authors:** S. Kutcher, H. Gilberds, C. Morgan, R. Greene, K. Hamwaka, K. Perkins

**Affiliations:** 1 Dalhousie University and the Izaak Walton Killam (IWK) Health Centre, 5850 University Avenue, PO Box 9700, Halifax, Nova Scotia B3K 6R8, Canada; 2 Farm Radio International, Ottawa, Canada; 3 Guidance, Counselling and Youth Development Centre for Africa, Lilongwe, Malawi

**Keywords:** Adolescents, Africa, Depression, educators, global mental health, knowledge, Malawi, mental health literacy, stigma

## Abstract

**Background.:**

Mental health literacy is foundational for mental health promotion, prevention, stigma reduction and care. Integrated school mental health literacy interventions may offer an effective and sustainable approach to enhancing mental health literacy for educators and students globally.

**Methods.:**

Through a Grand Challenges Canada funded initiative called ‘An Integrated Approach to Addressing the Issue of Youth Depression in Malawi and Tanzania’, we culturally adapted a previously demonstrated effective Canadian school mental health curriculum resource (the Guide) for use in Malawi, the African Guide: Malawi version (AGMv), and evaluated its impact on enhancing mental health literacy for educators (teachers and youth club leaders) in 35 schools and 15 out-of-school youth clubs in the central region of Malawi. The pre- and post-test study designs were used to assess mental health literacy – knowledge and attitudes – of 218 educators before and immediately following completion of a 3-day training programme on the use of the AGMv.

**Results.:**

Results demonstrated a highly significant and substantial improvement in knowledge (*p* < 0.0001, *d* = 1.16) and attitudes (*p* < 0.0001, *d* = 0.79) pertaining to mental health literacy in study participants. There were no significant differences in outcomes related to sex or location.

**Conclusions.:**

These positive results suggest that an approach that integrates mental health literacy into the existing school curriculum may be an effective, significant and sustainable method of enhancing mental health literacy for educators in Malawi. If these results are further found to be sustained over time, and demonstrated to be effective when extended to students, then this model may be a useful and widely applicable method for improving mental health literacy among both educators and students across Africa.

## Introduction

Globally, up to 14% of the burden of disease is attributable to mental illnesses, with the onset of most mental disorders occurring before the age of 25 (Patel *et al.* 2007; Prince *et al.* 2007). Youth in particular are at risk for the onset of mental disorders, which create the single largest disease burden in this population, and Depression is predicted to become one of the leading causes of disease burden globally in the next decades (World Health Organization, 2001, 2014). The prevalence of Depression in low and middle income countries (LMICs) is similar to that in developed countries; however, reliable data are unavailable in most countries in sub-Saharan Africa (Patel *et al.* 2007).

According to available research, Depression in Malawi is common. Udedi (2014) found a prevalence rate of about 30% in attendees of the Matawade Health Center in Zomba, whereas Kauye *et al.* (2014) reported a rate of 19% in attendees of other clinics. In a study of pregnant women and young mothers (many of whom are teenagers), Stewart *et al.* (2014) found rates of Depression ranging between 10.7% and 21.1%. Kim *et al.* (2014) report a Depression rate of 20% in adolescents attending HIV/AIDS clinics. These data are similar to those reported in Nigeria (Fatiregun & Kumapayi, 2014) and Kenya (Khasakhala *et al.* 2013) where in-school adolescent Depression rates have been found to be 21.2 and 26.4, respectively.

Given that substantial numbers of young people worldwide spend the majority of their time in school during adolescence, schools are a natural place to implement activities focused on mental health promotion, prevention and intervention (Kieling *et al.* 2011; Kutcher, 2011; McGorry *et al.* 2011). Mental health literacy is foundational for improving access to care and reducing stigma related to mental illness (Jorm *et al.* 1997; Reavley & Jorm, 2011; Jorm, 2012; Wei *et al.* 2013; Kutcher & Wei, 2014; Kutcher *et al.* in press) and was initially defined by Jorm as ‘knowledge and beliefs about mental disorders which aid their recognition, management and prevention’ (Jorm *et al.* 1997). Informed by recent developments in the evolving definition of health literacy (Institute of Medicine, 2004; Rootman & Gordon-El-Bihbety, 2008; Kanj & Mitic, 2009; World Health Organization, 2013) and cognizant of considerations related to mental health (Canadian Alliance on Mental Illness and Mental Health, 2008; Reavley & Jorm, 2011; Jorm, 2012; Wei *et al.* 2013; Kutcher & Wei, 2014; Kutcher *et al.* in press), this definition has now been expanded to include four components: (1) enhancing capacity to obtain and maintain good mental health; (2) enhancing understanding of mental disorders and their treatments; (3) decreasing stigma related to mental illness; (4) enhancing help-seeking efficacy (Kutcher & Wei, 2014; Kutcher *et al.* in press).

Global efforts to address mental health in schools were initiated by calls to action from international agencies such as the World Health Organization (WHO) and the United Nations Educational, Scientific and Cultural Organization (UNESCO) and have focused on introducing programmes into schools which address pro-social behaviours, mental health promotion, suicide prevention and specific mental disorders, such as Depression and Substance Use Disorders (Wei & Kutcher, 2012). However, a sustained positive impact of these programmatic interventions on mental health literacy has not been widely nor consistently demonstrated (United Kingdom Department for Education, 2011; Weare & Nind, 2011; Wei & Kutcher, 2012, Wei *et al.* 2013). More recently, interventions in Norway and Canada that have focused on addressing mental health literacy through school implemented curriculum have demonstrated positive results (Milin *et al.* 2013; Skre *et al.* 2013; Kutcher & Wei, 2014; Kutcher *et al.* in press, McLuckie *et al.* in press; Wei *et al.* in press). Available Canadian data have shown that providing teachers with a curriculum ready resource (the High School Mental Health Curriculum Guide) as well as training teachers on the effective classroom use of the Guide leads to sustained improvements in mental health literacy for teachers (Wei *et al.* 2012; Kutcher *et al.* 2013; Kutcher & Wei, 2014; Wei *et al.* in press).

This positive impact has also been extended to students. When teachers apply the Guide in their classrooms as part of usual school curriculum, significant (‘*p*’ values less than 0.001), substantial (‘*d*’ values demonstrating moderate or high impact) and sustained (improvements maintained over time in the absence of additional interventions) positive results are found in enhancing knowledge, decreasing stigma and improving health-seeking behaviours for secondary school students (Milin *et al.* 2013; Kutcher & Wei, 2014; McLuckie *et al.* in press). This evidence suggests that improving mental health literacy through curriculum integration may be an effective approach, addressing both teachers and students concurrently.

Despite this growing empirical evidence of the positive impact of integrated school-based curriculum approaches in Western countries, there is, to our knowledge, no evidence of the utility or impact of this approach in LMIC countries. Addressing youth mental health literacy needs in resource constrained environments is rife with challenges, which include but are not limited to: absence of child and youth mental health policies; inadequate funding for child and youth mental health care; lack of awareness among policy makers of the impact of mental disorders on young people; lack of mental health literacy among young people, educators, health providers and the general population (Patel *et al.* 2007; Kieling *et al.* 2011; Wei *et al.* 2012). To help address some of these issues, a novel programme linking improvements in mental health literacy among educators and youth to improved mental health care of adolescents in primary care was developed by a Canadian team of mental health, education and communications experts. Funded by Grand Challenges Canada, ‘An integrated approach to addressing the challenge of Depression among the youth in Malawi and Tanzania’ was initiated in 2012 in three regions of Malawi. This programme consists of four components: enhancing mental health literacy in teachers and students by applying the Guide resource as described above; training youth peer mental health educators; training primary health care providers to identify, diagnose and treat Depression; and creating and distributing a youth friendly radio programme that uses a variety of innovative technologies and formats including a soap opera programme and call-in show. This paper reports on a portion of one of these components. We sought to determine the impact of a training programme for educators on how to use a culturally adapted school mental health curriculum resource [the African Guide: Malawi version (AGMv)] on the mental health literacy of educators in the Lilongwe, Mchinji and Salima districts of central Malawi.

## The setting

The Republic of Malawi is one of the poorest countries in the world. According to the World Bank, over 50% of its population lives below the poverty line of less than $1.25 a day at 2005 international prices (The World Bank, 2014*a*, *b*). There are currently only four psychiatrists to serve the total population of 15.7 million, and no child and adolescent psychiatrists (The World Health Organization, 2011). There is also a paucity of other mental health care professionals, such as social workers, psychologists and psychiatric nurses. There are three psychiatric hospitals in the entire country, and these are institutions that mostly service individuals who live with the severest and most disabling mental illnesses. Mental health services targeted towards common mental disorders are scarce, as are mental health services specifically for adolescents. Furthermore, mental health promotion and programmes designed to target mental health literacy are uncommon and the focus tends to be on service delivery for the most severe mental disorders (Kavinya, 2011; Journalists for Human Rights, 2012; Kauye *et al.* 2014; Udedi, 2014).

In addition to the scarcity of services for common mental disorders, poor understanding of mental health and mental illness persists in Malawi, as illustrated in health care site-based studies (MacLachlan *et al.* 1995; Crabb *et al.* 2012). In 2013 cross-sectional survey of over 2000 adolescents conducted by the Grand Challenges Project team in central Malawi, 95% of the respondents attributed the cause of mental disorders to alcohol and illicit drug abuse, 92.8% to brain disease, 82.8% to spirit possession and 76.1% to psychological trauma (Farm Radio International, 2014). Attribution of mental disorders to drugs, alcohol and spiritual aspects has been shown to be one cause of discrimination and maltreatment towards people with mental disorders (Crabb *et al.* 2012). Thus, there is substantial opportunity to address youth mental health literacy in Malawi, thereby potentially enhancing knowledge about mental disorders and their treatments, promoting mental health, decreasing stigma and decreasing barriers to mental health care (Gureje & Alem, 2000; Saxena *et al.* 2007; Crabb *et al.* 2012; Kutcher & Wei, 2014).

Our programme is set in three districts of the central region of Malawi – Lilongwe, Mchinji and Salima. These sites are all urban/semi-urban, and all contain a number of schools of each classification type – religious, public, private, boarding, mixed gender and single gender. The target intervention sites were chosen due to their similarity to one another – they all share the same language, culture and average income of inhabitants, and all have a major urban centre surrounded by rural communities and villages.

## Methods

### The intervention

The intervention consisted of training educators on the use of the AGMv to determine if this would have a positive impact on their mental health knowledge and attitudes (stigma) related to mental health.

The Mental Health and High School Curriculum Guide is a mental health literacy resource that was initially developed in Canada, designed for use in junior high and secondary schools, and certified by Curriculum Services Canada, a pan-Canadian curriculum standards and evaluation agency (Curriculum Services Canada, 2014). It underwent extensive field-testing, and a training programme to assist teachers in learning how to apply the Guide in their classrooms was developed (Kutcher & Wei, 2013). This training programme is consistent with the approach that schools take when new curriculum for classroom use is introduced to teachers. Once teachers are trained on the content and use of the resource they apply it in their classrooms using their own teaching methods. Subsequent evaluations, cross-sectional research studies and a randomized control trial have all reported extensive and lasting improvements in mental health literacy for both teachers and students using this approach (Kutcher & Wei, 2014).

This resource was further enhanced for the current programme in Malawi and Tanzania by an educational module specifically focusing on adolescent Depression developed for this project. The module was derived from a Canadian Adolescent Depression training programme that has been nationally certified for continuing medical education by the Canadian College of Family Physicians (TeenMentalHealth.org, 2014*a*) and reported on by the Pan American Health Organization (Pan American Health Organization, 2012).

The Guide consists of a teachers' self-evaluation test, a teachers' mental health knowledge self-study study guide and six classroom ready modules containing: learning objectives, major concepts addressed, lesson plans, classroom activities and teaching resources. The six modules are: the stigma of mental illness; understanding mental health and wellness; information about specific mental illnesses; experiences of mental illness; seeking help and finding support; and the importance of positive mental health. The resource is available in hard copy or online (available at www.teenmentalhealth.org). The online version includes all of the core classroom teaching materials and also contains additional resources, such as animated videos, digital storytelling videos and supplementary materials for further study. The teachers' training programme includes an overview of mental health and mental disorders based on materials (available at: www.teenmentalhealth.org) and a detailed review of the Guide resource (TeenMentalHealth.org, 2014*b*).

The Guide was modified and adapted for use in Malawi by educators, Ministry of Health consultants and counsellors affiliated with the Guidance, Counselling and Youth Development Center for Africa (GCYDCA). The adaptors reviewed and modified the Guide materials and determined how the revised contents could be put into context for Malawi. Plans for translation of the Guide by technical experts are currently underway. The revised version of the Guide (AGMv) received the final review and sign-off from Dr Dixie Maluwa Banda, Professor of Education and Psychology at the University of Malawi and former consultant to the Ministry of Education of Malawi and Dr Kenneth Hamwaka, Executive Director of the GCYDCA (United Nations Educational, Scientific and Cultural Organization, 2001).

This intervention used a teach-the-teacher approach, in which trainers who were mental health professionals (one psychologist and one psychiatrist) or who had some background in mental health (four staff members from GCYDCA) were trained as a group on the use of the AGMv. These trainers then taught educators (teachers and youth club leaders in participating schools and youth clubs) on the content and classroom application of the AGMv. This paper provides the results of the training sessions delivered to educators on the use of the AGMv in Malawi in 2013.

A 3-day training workshop was provided to educators at each site. The training was conducted by the same trainers for each workshop and focused on the basic concepts of mental health and mental disorders (using material derived from: http://teenmentalhealth.org/learn/ and http://teenmentalhealth.org/toolbox/school-mental-health-teachers-training-guide-english/), a module by module review of the AGMv, and group participatory discussion of various possible teaching strategies for implementing the AGMv in school classrooms and youth club meetings. Training sessions were provided to a total of 218 teachers and youth club leaders (121 male, 96 female and 1 gender not provided). Using co-facilitation, trainees were divided into small groups. Each group rotated facilitators for each module of the training guide until all six modules were completed.

### Study design

In order to evaluate the impact of the intervention on educators, a repeated measures/within participants study design was employed. Participants' knowledge about and attitudes towards mental illness were measured at the beginning and again at the completion of a 3-day training period. To assure anonymity, participants were asked not to provide any identifying information on the test materials, and anonymous identifiers, such as month of birth, favourite food and mother's first name, were used to link participants' pre- and post-training responses. The training was conducted and data obtained in 2013.

### Questionnaire and outcome measures

An evaluation of mental health knowledge and attitudes was conducted using previously validated (Kutcher *et al.* 2013) written pre- and post-tests that were reviewed for cultural appropriateness by GCYDCA staff. The pre-tests were completed by trainees immediately prior to the start of the training session and the post-tests immediately following. The knowledge tests consisted of 30 questions (Cronbach's *α* = 0.638) accompanied by ‘true’, ‘false’ and ‘I don't know’ options. Participants were instructed to choose only one option per question and were encouraged to mark ‘I don't know’ rather than guessing. Eight questions (Cronbach's *α* = 0.549) were used to measure attitudinal change using a seven-point Likert Scale, ranging from ‘strongly disagree’ to ‘strongly agree’. A total positive attitude score out of 56 was calculated.

### Analysis

PASW Statistics 17 was used to conduct paired *t* tests to evaluate the differences in knowledge and attitude scores at baseline and immediately following the intervention. Differences in improvements in knowledge and attitudes based on sex were evaluated using an independent samples test (*t* test). Knowledge and attitude questions were examined individually to ascertain which specific questions had scores that improved following the training.

## Results

### Participant characteristics

Workshop participants were teachers and youth club leaders selected by the Ministry of Education from both primary and secondary schools (see Table 1). Youth clubs are out-of-school groups typically populated by school drop-outs, or young people who have recently completed secondary school but are unemployed (average age is 20–30 years old). They were initially established in Malawi by non-governmental organizations for young people to disseminate health information about HIV/AIDS to their peers and community members through role plays, songs and poems. These groups have expanded to incorporate a number of other education and health-related objectives. They often meet weekly, biweekly or monthly to socialize and develop outreach activities. Youth club leaders are responsible for the oversight and management of the clubs.

**Table 1.** Participant gender distribution by region

Region	Male	Female	Total	Pearson χ^2^
Lilongwe	33 (53.2%)	29 (46.8%)	62	*p*=0.772
Mchinji	33 (54.1%)	28 (45.9%)	61	
Salima	55 (58.5%)	39 (41.5%)	94	
All regions (total^a^)	121	96	217	

^a^One gender from Lilongwe unknown.

Of the total of 218 participants, all of the knowledge tests and 194 of the attitude tests (24 participants did not complete both the pre and post-attitude scales) could be matched for statistical analysis. As is common in Malawi, teachers had a wide variety of educational backgrounds and taught a broad range of subjects, including Mathematics, Social Studies, Chichewa, English, Geography and Agriculture. Youth club leaders selected from out-of-schools clubs were located in the community and do not teach any subjects.

### Knowledge results

**Fig. 1.**
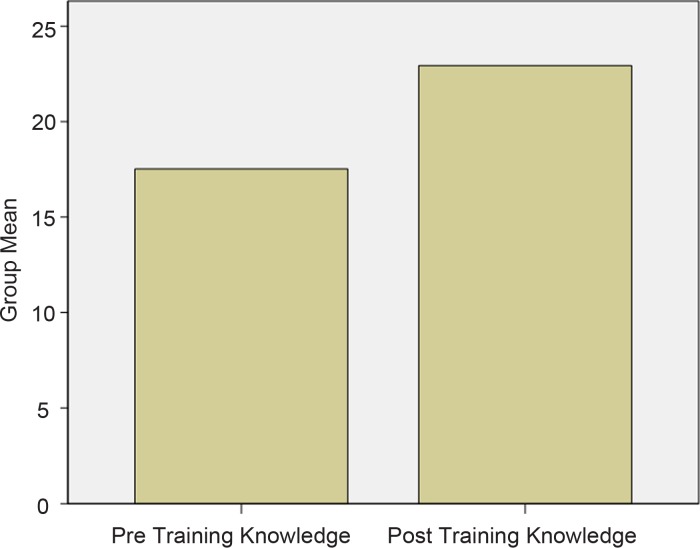
Mean Scores for Educators' General Mental Health Knowledge.

Outcomes of the knowledge assessment survey show that prior to the training, educators (*n* = 218) correctly answered an average of 58.3% (*M* = 17.5, s.d. = 4.07) of the 30 questions about mental health, mental illness and Depression. This improved to 76.3% (*M* = 22.94, s.d. = 2.89), immediately following completion of the training programme. This change is highly statistically significant, *t*(217) = 17.10, *p* < 0.0001 (see Fig. 1). This analysis demonstrated an effect size of *d* = 1.16. This large effect size indicates that the training had a substantial impact on educators knowledge acquisition.

### Attitude results

**Fig. 2.**
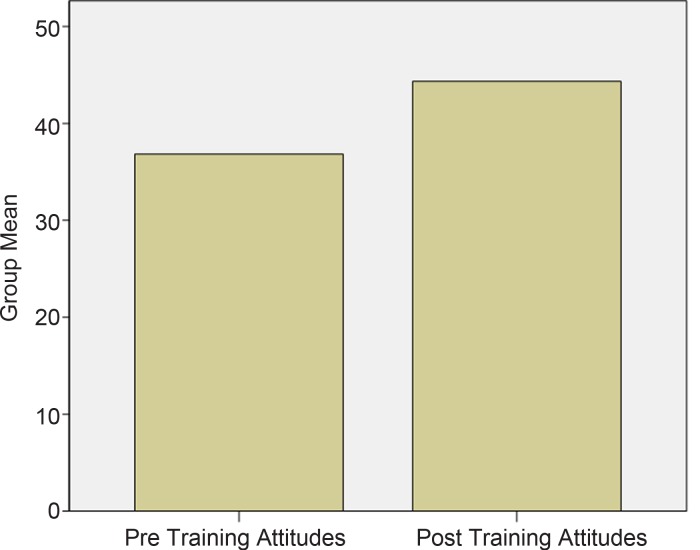
Mean Scores for Educators' Attitudes towards Mental Illness.

The educators demonstrated moderately positive attitudes towards mental illness at baseline (*M* = 36.84, s.d. = 8.36). This increased after training (*M* = 44.33, s.d. = 7.59). These results were highly statistically significant, *t*(193) = 11.04, *p* < 0.0001 (see Fig. 2), illustrating the positive impact that the training had on attitudes towards mental health. The effect size (*d* = 0.79) indicates a large increase in educators' positive attitudes and a decrease in stigmatizing attitudes.

There were no significant differences in score improvements in either knowledge or attitudes by sex or location (all analyses *p* > 0.05). An item-by-item analysis of change on the knowledge test showed significant improvement on 28 of 30 questions (correct responses increasing by at least 2.7% and at most 41.3% of respondents). An item-by-item analysis of change on the attitudes test showed significant improvement on all items (average improvements ranging from 0.45 to 1.63 on individual questions).

## Discussion

The results reported herein clearly demonstrate a highly significant and substantial positive impact on the mental health literacy of educators who received training on the use of the African Guide Malawi version. These improvements occurred, not as a result of a specific mental health training intervention (such as a mental health literacy course) directed towards educators, but rather as a ‘by-product’ of training educators on how to use a mental health curriculum resource (the AGMv) in their classrooms. These results were found independent of sex or location.

The importance of embedding mental health into existing health and education policy, planning and applications in low-income countries is well recognized (Hendren *et al.* 1994; World Health Organization Expert Committee on Comprehensive School Health Education and Promotion, 1997; World Health Organization, 2003; Rowling & Weist, 2004; Jacob *et al.* 2007; Patel *et al.* 2007; Prince *et al.* 2007; Saxena *et al.* 2007; Wei *et al.* 2011; Crabb *et al.* 2012; Wei *et al*, 2012*a*). Reports have time and again insisted that interventions should be effective, financially realistic, contextually specific and embedded within existing health and education systems to increase uptake of use and promote sustainable application that will not vanish when project funding ends Kutcher (2011); World Health Organization, 2014; Kutcher *et al.* in press). The intervention discussed in this paper incorporated all of these elements from the outset. In this study, we report that this approach has demonstrated a positive impact on African educators' own mental health literacy. This is a positive first step that needs to be further evaluated in subsequent research to determine if this approach will translate into improved African student mental health literacy when the resource is actually applied by trained educators in the usual school setting.

This approach to enhancing mental health literacy for educators in a pedagogically contextualized manner has a number of strengths not found in non-curriculum-based approaches that provide mental health information to educators, such as mental health first aid (Jorm *et al.* 2010). First, the positive improvements in educators' mental health literacy were realized using a methodology consistent with usual education pedagogy. Educators were provided training on the use of a classroom mental health literacy resource (the AGMv) so that they would be able to apply it in their own classrooms, using their own professional skills. As such, this replicates the way that educators all over the world prepare themselves for teaching students. This approach is thus both familiar to educators and administrators alike and relatively easy to implement in educational settings. It does not require substantial additional programme development, implementation and maintenance resources. It both builds on and enhances existing competencies of teachers. For this reason, it can potentially be applied in different settings with similar results. For example, in this study there were no significant differences found in outcomes by sex or educator location. Indeed similar results have been reported in widely dissimilar settings as demonstrated when comparing these findings with data from different Canadian provinces (Wei *et al.* 2012; Kutcher & Wei, 2013, 2014).

Second, this pedagogically contextualized approach may be familiar and thus more acceptable to education bureaucracies. For example, based on this approach and these results, the AGMv is being reviewed by the Malawi Ministry of Education as a possible component of the national school curriculum. It was also recognized by the Ministry of Health as an important component of the reform of mental health policy and plans for Malawi [Dr Hamwaka, personal communication and Dr Mugomba (Ministry of Health), personal communications]. While this result is promising, further evaluations of how educational policy makers will respond to and apply this contextualized mental health literacy for educators approach in LMCs is needed. We are currently engaged in replication of this approach in Tanzania. Comparing and contrasting the outcomes in these two different African settings will allow for better understanding of the opportunities and barriers that this approach faces vis-à-vis acceptance into education policies, plans and implementations.

Third, training pre- and post-test results demonstrate significant and substantial improvements in educators' mental health literacy regardless of sex or location. These results suggest that scale-up of this approach may prove to be an effective method for enhancing the mental health literacy of educators within the rest of Malawi. This potential, however, remains to be determined with further research.

Fourth, teaching educators to use a classroom mental health curriculum resource (the AGMv) not only improved their own mental health literacy, but also provided them with a resource that they can now use in their classrooms to educate their students. Thus, the potential exists for the widespread improvement of the mental health literacy of young people in classrooms. Such an approach fits well within a ‘whole school’ framework (Wells *et al.* 2003; Jané-Llopis *et al.* 2005; Barry *et al.* 2007) directed towards improvement of students' mental health. While this outcome has yet to be tested in the Malawian context, recent studies in Canada have clearly demonstrated substantial improvements in students' mental health literacy using this approach (Kutcher & Wei, 2014). This approach would also be complementary to various other school mental health initiatives currently underway in some high-income countries. For example, it would potentially enhance the current UK school mental health strategic directions (Crowe, 2014). In Canada, the national child and youth mental health framework has identified the need for enhancing school-based mental health interventions (Mental Health Commission of Canada, 2010) and two Canadian provinces are currently embedding this MHL approach into grade nine curriculum while five others have active applications ongoing in both junior high and secondary schools.

### Limitations

Despite its successes in improving educators' mental health literacy by enhancing knowledge and decreasing stigma, this study has certain limitations. The pre/post-design carries with it inherent problems compared to a controlled experiment or a design that uses a control group. It is not possible to fully attribute the improvements made by the teachers over the course of this intervention to the intervention itself. However, the timeline of the assessments – immediately prior to and immediately after the intervention – make other explanations for the observed improvements unlikely.

Nonetheless, the short-term follow-up limits understanding of the long-term impact of this intervention, and the study design cannot evaluate improvements in educators' mental health literacy retention over time or the impact of their classroom application of this resource. It is not yet known if educators trained in the African Guide Malawi version will retain improved knowledge and attitudes over time, nor if they will be able to successfully use the resource in their classrooms to improve mental health literacy in their students. These studies have yet to be conducted, but we are currently addressing these issues in research underway in Malawi.

Furthermore, these early positive results may not stand the test of real life application into educational policy, plans and successful implementation. The challenges to successfully addressing youth mental health in low-income settings are well known and it remains to be seen if this pedagogically contextualized approach may be successfully embraced and delivered throughout Malawi and elsewhere in Africa.

## Conclusion

This study evaluated the immediate effects of a mental health literacy resource (the African Guide Malawi version) educator training programme and found that this approach improved mental health literacy among educators simply by teaching them how to use the resource in the classroom setting. According to the results obtained, the intervention produced a highly significant and substantial positive impact on the mental health literacy of educators involved in the programme. This approach builds on and expands the effectiveness of improving the mental health literacy of educators through a pedagogically familiar model that could potentially be integrated into most school settings in which there is a willingness and ability to address mental health literacy. This embedded mental health literacy approach addresses mental health promotion, stigma reduction and understanding of mental illnesses and mental health care, thereby integrating many aspects that are often addressed separately in the school setting. Further, this approach – training educators on the application of an inexpensive and teacher friendly resource and embedding mental health literacy into existing curriculum in usual classrooms and youth club meetings – holds potential for widespread applicability and merits further exploration and evaluation.

Further research into the application of this approach in addressing mental health literacy for educators and students in Malawi and other parts of sub-Saharan Africa is now underway. Finally, given that this approach of embedding MHL into teacher delivered school curriculum may have global applicability, studies are underway in a number of other countries, including high-income locations such as Canada.
